# Outcomes and Strategies to Support a Treat-to-target Approach in Inflammatory Bowel Disease: A Systematic Review

**DOI:** 10.1093/ecco-jcc/jjz131

**Published:** 2019-08-12

**Authors:** Jean-Frédéric Colombel, Geert D’haens, Wan-Ju Lee, Joel Petersson, Remo Panaccione

**Affiliations:** 1 Inflammatory Bowel Disease Center, Icahn School of Medicine at Mount Sinai, New York, NY, USA; 2 Amsterdam University Medical Centers – Inflammatory Bowel Disease Unit, University of Amsterdam, Amsterdam, The Netherlands; 3 Global Gastroenterology, AbbVie, North Chicago, IL, USA; 4 Inflammatory Bowel Disease Clinic, Cumming School of Medicine, University of Calgary, Calgary, AB, Canada

**Keywords:** Endoscopy, inflammatory bowel disease, outcomes

## Abstract

**Background and Aims:**

Management of Crohn’s disease and ulcerative colitis has typically relied upon treatment intensification driven by symptoms alone. However, a ‘treat-to-target’ management approach may help to address underlying inflammation, minimise disease activity at early stages of inflammatory bowel disease, limit progression, and improve long-term outcomes.

**Methods:**

A systematic literature review was conducted to identify data relevant to a treat-to-target approach in inflammatory bowel disease, published between January 1, 2007 and May 15, 2017.

**Results:**

Consistent with recommendations of the Selecting Therapeutic Targets in Inflammatory Bowel Disease [STRIDE] working group, studies have investigated factors influencing the achievement of both endoscopic and histological mucosal healing and patient-level outcomes in inflammatory bowel disease [IBD]. Histological healing and biomarker levels have also been shown to be modifiable outcomes. Although there is a lack of prospectively derived evidence validating mucosal healing as a treatment target, data are emerging to suggest that targeting mucosal healing or inflammation rather than symptoms may be cost-effective in some settings. The review highlighted several strategies that may support the implementation of a treat-to-target approach in IBD. The prospective randomised CALM study demonstrated how tight control [whereby treatment decisions are based on close monitoring of inflammatory biomarkers] leads to improvements in endoscopic and clinical outcomes. The review also considered the influence of coordinated care from a multidisciplinary team and patient engagement with improved adherence, as well as the role of therapeutic drug monitoring in inflammatory bowel disease management.

**Conclusions:**

A treat-to-target strategy may impact on disease progression and improve outcomes in inflammatory bowel disease. Prospective studies including long-term data are required to ensure that the most appropriate targets and strategies are identified.

## 1. Introduction

Crohn’s disease [CD] and ulcerative colitis [UC] are progressive inflammatory disorders of the gastrointestinal tract which can lead to serious complications and disability if not optimally treated.^1–3^ The conventional approach to treatment of these conditions—collectively termed inflammatory bowel disease [IBD]—has focused on control of symptoms using a step-up pharmacological intervention strategy, with progressive intensification of therapy and/or surgical resection as the disease worsens or therapy fails to control symptoms.^4–7^ Treatment options include non-specific therapies such as different formulations of the anti-inflammatory agent 5-aminosalicylic acid [5-ASA], glucocorticosteroids, immunomodulators, and more specific biologic therapy such as anti-tumour necrosis factor-alpha antibodies [anti-TNFα].^5^ More recent additions include anti-integrins, anti-interleukin 12/23 agents, and the JAK inhibitor tofacitinib.^5,8^ Surgical resection is usually reserved for patients with complications or those with intractable disease.

However, it is becoming increasingly apparent that treatment strategies aimed purely at controlling symptoms do not alter the course of these disorders, as they fail to induce repair of the inflammatory lesions.^9,10^ Even after surgical resection, especially in CD, the disease can often recur. As is the case for rheumatoid arthritis, strategies for the management of IBD have changed substantially in recent years, with a move towards preventing disease progression and improving long-term outcomes for patients.^4^ Newer management strategies focus on control of both symptoms and inflammation—demonstrated by clinical outcomes such as mucosal healing [both endoscopic and histological] and fistula repair—rather than control of symptoms alone.^10^ Mucosal healing in patients with early-stage IBD often predicts sustained remission^11^ and may be associated with reduced hospitalisation and surgery.^12^

Recognition of the importance of minimising disease activity at an early stage in IBD has led to the consideration of a ‘treat-to-target’ [T2T] management approach. Treatment targets are defined with the aim of improving outcomes and reducing the risk of end-organ damage such as the development of stricture, fistula, or functional impairment. Such strategies have been adopted for various chronic disorders including rheumatic conditions, vascular medicine, and metabolic disorders such as diabetes mellitus.^13,14^ A T2T approach is, however, a collaborative approach between the physician and the patient. It involves identifying an appropriate target, selecting initial therapy according to the risk of disease progression, measuring baseline characteristics of disease, monitoring progress, and optimising therapy to reach the agreed goal.

We report here a systematic literature review designed to explore the data available to support a T2T approach in IBD, using various strategies. In the first instance, this involves an assessment of the appropriateness of various potential clinical targets, including symptoms, biomarkers, and endoscopic-based outcomes and quality of life measures, as well as levels of biomarkers or serum drug concentrations. Data relating to the impact of T2T management on economic outcomes are also considered. The remainder of the review covers data supporting a range of strategies supporting the implementation of a T2T approach to eventually improve outcomes. These strategies include: achievement of tight disease control; the involvement of a multidisciplinary team; adherence of patients to treatment regimens; and therapeutic drug monitoring.

## 2. Methods

The systematic literature search was designed to ascertain the impact of a T2T IBD management approach on short-term clinical outcomes [patient and health care professional [HCP]-centred outcomes], long-term benefits [e.g. rates of hospitalisation/surgery], and costs.

The search terms for the literature search comprised three elements; publications were required to include terms relating to: [i] T2T or related tools/care strategy; [ii] IBD; and [iii] patient/HCP/cost outcomes [search terms are detailed in the Supplementary Appendix, available as Supplementary data at *ECCO-JCC* online].

Searches were conducted in Embase® and Medline® for articles published between 1 January 2007 and 15 May 2017. In addition, conference proceedings from Digestive Disease Week [DDW; Chicago, USA, May 6‑9, 2017] were hand-searched to ensure that emerging data were included.

Articles identified through these searches underwent a two-stage screening process. Titles and abstracts were scanned for relevance to the clinical question being investigated. Second, full-text copies of relevant articles were obtained and reviewed against the inclusion criteria [Supplementary Appendix]. Any duplicate articles, or those considered to be of insufficient quality on the basis of population, design, or relevance, were excluded. Articles were required to report primary data; systematic reviews could be included at the full-text screening stage in order to check their bibliographies for relevant publications, but were themselves excluded.

Data were extracted from included articles by one reviewer into a data extraction table and checked for accuracy by a second reviewer. Where available, data were extracted relating to: study characteristics [design, region]; participant characteristics [population: CD/UC/mixed], length of disease, sample size]; baseline characteristics [age, gender, treatment history, disease severity]; treatment [comparators, T2T elements]; and outcomes after management [clinical scores, hospitalisation, surgery, symptoms, 

remission].

## 3. Results

**Figure 1.**
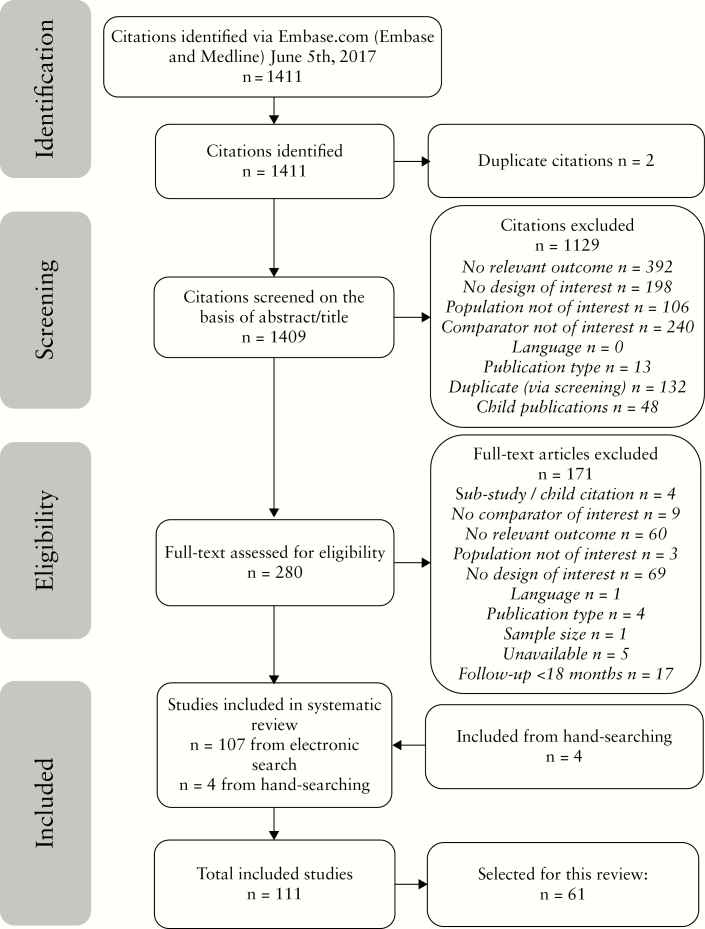
PRISMA diagram showing identified, screened and included articles. In addition, five articles published since the search was conducted were identified by the authors as relevant to the review topic.^75–79^

In total, 1409 articles were screened, 280 were assessed for eligibility [full-text], and 111 were included in the data extraction and analysis [Figure 1]. Of these, 61 articles were identified to support the focus on the association between a T2T approach and outcomes in this review [Table 1].^9,15–74^ In addition, five articles published since the search was conducted were identified by the authors as relevant to the review topic.^75–79^ To provide context to the discussion of these data, some additional background material was identified by the authors.^11,13,80–91^

**Table 1.** Summary of publications identified by literature review [61] and by authors *et al.* [5] to support review.

Publication	N	Condition	Study design	Class of evidence^a^	Main outcomes
Colombel J-F, 2017^16^	244	CD	Open-label phase 3 RCT	I	Endoscopic and deep remission outcomes
Colombel J-F, 2017^17^	244	CD	Open-label phase 3 RCT	I	Adverse outcomes [hospitalisations, surgeries]
Colombel J-F, 2013^36^	778	CD	Analysis of data from RCT and open-label extension	I	QOL [SF-36, IBDQ]
Colombel J-F, 2018^b^^75^	244	CD	Open-label phase 3 RCT	I	Clinical and endoscopic outcomes, safety
de Jong MJ, 2017^b^^76^	909	IBD	Pragmatic RCT	I	Number of outpatient visits, patient-reported quality of care, safety
D’Haens G, 2018^b^^78^	122	CD	Double-blind RCT	I	Sustained corticosteroid-free clinical remission
Elkjaer M, 2010^42^	333	UC	RCT	I	Feasibility of ‘constant care’ approach, influence on patients’ compliance, knowledge, QOL, disease outcomes, safety and health care costs
Hueppe A, 2014^41^	514	CD/UC	RCT	I	Health-related QOL, social participation
Khanna R, 2015^22^	1982	CD	Open-label cluster RCT	I	Proportion of patients in corticosteroid-free remission; adverse outcomes
Lasson A, 2015^34^	91	UC	Prospective, RCT	I	Relapse rate
Rutgeerts PJ, 2010^35^	62	CD	Post-hoc analysis of randomised, placebo-controlled study	I	CDAI scores and clinical remission status
Steenholdt C, 2015^51^	69	CD	RCT, single-blind, multicentre study	I	Long-term economic outcomes
Steenholdt C, 2014^52^	69	CD	RCT, single-blind, multicentre study	I	Response rate [CDAI], accumulated treatment-related costs
Vande Casteele N, 2015^50^	263	CD/UC	RCT	I	Clinical and biochemical remission
Bougen G, 2014^9^	67	CD	Retrospective, chart review	II	Mucosal healing [defined as the absence of any ulcers in any segment of the gastrointestinal tract during the endoscopic procedure]
Bougen G, 2014^15^	60	UC	Retrospective, chart review	II	Mucosal healing (defined as unremarkable findings including absence of any type of friability [even mild] and with possible remaining slight and patchy loss of vascular pattern, or erythema from inactive disease equivalent to a zero Mayo endoscopic subscore)
Burke K, 2013^60^	107	UC	Prospective observational cohort study	II	Long-term relapse rates and healthcare costs
Carter CT, 2011^61^	638	CD	Retrospective claims analysis	II	Adherence rates
Carter CT, 2012^62^	448	CD	Retrospective claims analysis	II	Adherence rates, hospitalisations, length hospital stay, inpatient costs
Chavannes M, 2016^46^	188	IBD	Single-centre retrospective cohort study	II	Serum levels of infliximab
Click BH, 2016^30^	1600	IBD	Registry review	II	Financial health care use and disease activity
Cook PF, 2010^71^	524	UC	Structured patient interviews	II	Impact of telephone nurse counselling on adherence
David G, 2014^54^	21076	IBD	Analysis of data from commercial databases	II	Adherence rates
D’Incà R, 2015^72^	449	CD/UC	Patient survey	II	Adherence, quality of life, illness intrusiveness
Debanjali M, 2009^63^	1693	UC	Analysis of data from claims database	II	Impact of medication adherence on costs and all-cause health care use
Feagan BG, 2014^64^	945	CD	Analysis of data from claims database	II	Health care costs by adherence status
Hodgkins P, 2012^65^	400	UC	Discrete-choice experiment	II	Differences in patient treatment preferences based on self-reported adherence
Kane S, 2008^66^	4313	UC	Analysis of data from claims database	II	Adherence, health care costs
Kane SV, 2009^67^	571	CD	Analysis of data from claims database	II	Adherence, health care use, costs
Lachaine J, 2011^68^	1681	UC	Retrospective prescription and medical claims analysis	II	Adherence, health care use, costs
Little RD, 2016^18^	52	IBD	Retrospective observational study	II	Clinical response [biomarker and physician global assessment]
Michels S, 2014^59^	173	CD	Analysis of data from claims database	II	Health care costs by different adherence thresholds
Mitra D, 2012^58^	1693	UC	Retrospective analysis of insurance claims	II	Adherence, all-cause costs and health care use
Orlaith K, 2016^47^	312	IBD	Single-centre retrospective study	II	Endoscopic remission
Papamichail K, 2016^48^	43	UC	Single-centre retrospective study	II	Short-term mucosal healing [defined as Mayo endoscopic sub-score of ≤1, assessed at Weeks 8–14, with a baseline sub-score of ≥2]
Paul S, 2013^49^	52	CD/UC	Prospective observational study	II	Mucosal healing [defined as faecal calprotectin <250 μg/g stools in CD and by an endoscopic Mayo score of 0 or 1 in UC]
Poillon L, 2018^b^^77^	226	CD/UC	Retrospective single-centre follow-up of ^50^	II	Long-term outcome data [IBD-related hospitalisation, abdominal surgery, and systemic steroid use], continued use of infliximab, trough concentrations
Qiu Y, 2016^45^	272	CD	Retrospective, observational cohort study	II	Mucosal healing [defined as a score of 0–2 using an endoscopic score system]
Ramos Rivers CM, 2014^33^	1925	IBD	Prospective observational registry study	II	Patients calling out of hours
Ray I, 2013^29^	650	CD	Single-centre, retrospective study	II	Severity of depression, pattern of outpatient service use, costs
Regueiro M, 2016^37^	308	CD/UC	Observational study of patients enrolling in patient-centred medical home	II	QOL [SIBDQ], health care resource use [ER visits, hospitalisations]
Regueiro M, 2016^38^	108	CD/UC	Observational study of patients enrolling in patient-centred medical home	II	IBD activity [UCAI and CD HBI], QOL [SIBDQ], depression [PHQ9]
Sandborn W, 2015^20^	804	UC	Retrospective chart review, adalimumab vs infliximab	II	Real-world effectiveness [symptoms and disease activity] and resource use [hospitalisation and surgery rates]
Schechter A, 2015^31^	115	UC	Chart review	II	Sustained steroid-free remission, colectomy
Schifrien B, 2013^69^	3406	CD	Retrospective claims database analysis	II	Adherence, health care costs
Selinger C, 2012^55^	50	UC	Face-to-face structured interview with patients	II	Preferred mode of information delivery, thresholds for adherence
Seth N, 2014^32^	542	CD	Prospective registry study	II	Persistent abdominal pain
Severs M, 2016^56^	2612	CD/UC	Prospective observational cohort study	II	Factors associated with non-adherence, changes in adherence and associated disease outcomes
Severs M, 2016^57^	2612	CD/UC	Prospective observational cohort study	II	Impact of medication adherence on the disease course, health care costs and health-related QOL
Taks M, 2017^43^	33	IBD	Single-centre evaluation of treatment algorithm	II	Remission rates, drug costs
Van Deen WK, 2016^39^	98 [plus 293 controls]	IBD	Observational control-matched study	II	IBD-specific outcomes including medication use, office visits, IBD-specific tests, ED visits, and hospitalisations
Van Deen WK, 2016^40^	98 [plus 293 controls]	IBD	Observational control-matched study	II	IBD-specific outcomes including medication use, office visits, IBD-specific tests, ED visits, and hospitalisations
Wan GJ, 2014^70^	1646	IBD	Database analysis	II	Adherence, health care costs
Yarur AJ, 2017^28^	117	CD	Cross sectional study	II	Fistula healing/closure, mucosal healing [defined as the absence of ulcerations ≥5 mm in the colon and terminal ileum]
Zittan E, 2016^27^	60	CD	Chart review/patient interview	II	Clinical and endoscopic remission
Ananthakrishnan AN, 2012^24^		CD	Decision analytic model comparing treatment strategies	III	Clinical response, QALYs, ICER, NNT to prevent surgery/hospitalisation
Ananthakrishnan AN, 2013^25^		CD	Decision analytic model comparing treatment strategies	III	Clinical response, QALYs, ICER, NNT to prevent surgery/hospitalisation
Mallow P, 2013^21^		UC	Cost-effectiveness modelling based on data from RCT	III	Cost per clinical response and NNT for clinical response
Panaccione R, 2017^b^^79^	244	CD	Cost-effectiveness modelling based on data from RCT	III	Remission rates, CD-related hospitalisations, adalimumab injections, direct medical costs, QALYs, ICER, NMB
Saini SD, 2012^23^		UC	Markov cohort model	III	Cost utility outcomes
Thwaites PA, 2016^44^		IBD	Economic modelling	III	Costs of intestinal ultrasound and colonoscopy to the patient and the hospital
Van Deen W, 2015^26^	411	CD/UC	Validation of a 4-question smartphone app to monitor IBD activity	III	Clinical disease activity indices, QOL, endoscopic score
Van Deen W, 2014^74^	642	IBD	Developing and testing multidisciplinary care programmes for IBD patients—case scenarios	III	Clinical disease activity indices [HBI for CD and partial Mayo score for UC]; quality of life scores; health care expenditures
Van Deen W, 2014^19^	642	IBD	Developing and testing multidisciplinary care programmes for IBD patients—case scenarios	III	Clinical disease activity indices [HBI for CD and partial Mayo score for UC]; quality of life scores; health care expenditures
Velayos FS, 2013^53^		CD	Decision analytical model	III	Cost per QALY gained
Yen L, 2013^73^		UC	Budget impact model	III	Direct costs

CD, Crohn’s disease; CDAI, Crohn’s Disease Activity Index; ED, emergency department; ER, emergency room; HBI, Harvey-Bradshaw Index; IBD, inflammatory bowel disease; IBDQ, Inflammatory Bowel Disease Questionnaire; ICER, incremental cost effectiveness ratio; NMB, net monetary benefit; NNT, number needed to treat; QALY, quality-adjusted life year; QOL, quality of life; PHQ9, Patient Health Questionnaire-9; RCT, randomised controlled trial; SF-36, Short-Form 36; SIBDQ, Short Inflammatory Bowel Disease Questionnaire; UC, ulcerative colitis; UCAI, Ulcerative Colitis Activity Index.

^a^Classification as follows: I, prospective RCT; II, observational/database study; III, modelling/other.

^b^Five publications published since the search was conducted; identified by the authors as relevant to the review topic.

### 3.1. Impact of T2T on clinical short-term and long-term outcomes

The ‘Selecting Therapeutic Targets in Inflammatory Bowel Disease’ [STRIDE] International Organization for the Study of Inflammatory Bowel Diseases [IOIBD] working group sought to achieve international expert consensus on appropriate evidence-based treatment targets for IBD which could be used in T2T strategies in routine clinical practice.^13^ The group recommended that the primary therapeutic target in both UC and CD should be the composite endpoint of both clinical/patient-reported outcome [PRO] and endoscopic remission. For UC, clinical/PRO remission was defined as resolution of rectal bleeding and diarrhoea/altered bowel habit, with endoscopic remission described as a Mayo endoscopic subscore of 0–1. In the case of CD, clinical/PRO remission was identified by resolution of abdominal pain and diarrhoea/altered bowel habit, with endoscopic remission defined as resolution of ulceration at ileocolonoscopy, or resolution of findings of inflammation on cross-sectional imaging in patients who cannot be adequately assessed with ileocolonoscopy. STRIDE recommended that clinical/PRO remission should be assessed at a minimum of 3 months during active disease for both UC and CD, and endoscopic remission should be assessed at 3-monthly intervals during active disease for UC, or at 6–9-monthly intervals for CD.^13^ Adjunctive targets/measures for UC and CD were identified as histological remission and biomarker remission (normal C-reactive protein [CRP] and calprotectin), respectively, though these were not recommended as targets owing to a lack of evidence.^13^

The focus on mucosal healing in the STRIDE recommendations was supported by indirect evidence of its association with outcomes such as reduced risk of relapse, fewer surgeries, fewer hospitalisations, and successful steroid tapering.^11,13,80–87^ Our literature review revealed that most of the support for a T2T approach with the aim of achieving mucosal healing has been derived from retrospective studies.

Two single-centre retrospective chart review studies demonstrated that modification of treatment based on close endoscopic monitoring was associated with mucosal healing in patients with CD^9^ and UC,^15^ respectively. The CD study included 67 patients with a confirmed diagnosis and evidence of ulcers at an initial endoscopic procedure, who underwent at least two consecutive endoscopic procedures during the study period [in fact, 31% had three consecutive endoscopic procedures and 9% had four].^9^ Mucosal healing was defined as the absence of any ulcers in any segment of the gastrointestinal tract during the endoscopic procedure, and was achieved by 19.4%, 41.8%, and 50.7% patients at 24 weeks, 52 weeks, and end of follow-up [median 62 weeks], respectively. Two factors were significantly associated with mucosal healing: repeated endoscopic procedures within 26 weeks [versus more than 26 weeks] from the previous endoscopic procedure (hazard ratio [HR], 2.35; 95% confidence interval [CI], 1.15–4.97; *p* = 0.019) and adjustment of medical therapy [introduction or switch of immunosuppressives; introduction, optimisation, or switch within the class or out of the class of biologics; or changes in both immunosuppressives and biologics] when mucosal healing was not observed [HR, 4.28; 95% CI, 1.9–11.5; *p* = 0.0003]. Of the total 72 adjustments in medical therapy, 12.5% were made in the absence of clinical symptoms.^9^ Similar findings were reported in the UC study, in which 60 patients underwent at least two consecutive endoscopic assessments [43%, 13%, and 0.03% had 3, 4, and 5 assessments, respectively].^15^

 In this study, mucosal healing was defined as unremarkable findings including absence of any type of friability [even mild], with possible remaining slight and patchy loss of vascular pattern or erythema from inactive disease equivalent to a zero Mayo endoscopic subscore. The proportions of patients with mucosal healing at Weeks 26, 52, and 76 were 31.1%, 46.6%, and 53.3%, respectively. This study also evaluated histological healing, defined as the absence of inflammatory infiltrate but with possible remaining disturbance of the architecture of the mucosa; the proportions of patients achieving this target were 25%, 35.4%, and 50% at the three time points, respectively. As was the case in the CD study, adjustment in medical therapy in case of persistent inflammatory activity was associated with mucosal healing [HR, 9.8; 95% CI, 3.6–34.5; *p* < 0.0001] and—in this case—also with histological healing [HR, 9.2; 95% CI, 3.4–31.9; *p* < 0.001]. Of the total 51 adjustments in medical therapy, 15.6% were made in the absence of clinical symptoms.^15^

These data are supported by the findings of a retrospective observational cohort study of 272 patients with CD, in which disease management was based on serial endoscopic findings. The cumulative probability of mucosal healing [defined as a score of 0–2 using an endoscopic score system] increased from 10% at 6 months to 77.6% after 60 months.^45^ Factors independently associated with mucosal healing included frequent endoscopic procedures [separated by intervals of fewer than 26 weeks; HR, 1.56; 95% CI, 1.05–3.39] and adjustment of medical therapy when healing was not achieved [HR, 2.07; 95% CI, 1.26–2.33].

The open-label ‘Randomised Evaluation of an Algorithm for Crohn’s Treatment’ [REACT] trial was an assessment of an intervention strategy for IBD, focused on a target of clinical remission.^22^ In this cluster randomisation trial, 41 community gastroenterology practices were assigned to an algorithm of treatment escalation to early combined immunosuppression with a TNF antagonist and antimetabolite based on symptoms [Harvey‑Bradshaw Index remission and no corticosteroid use] or conventional management.^22^ It is worth noting that although the study was designed to assess impact of protocolised drug escalation, patients had an average disease duration of more than 12 years. There was no significant between-group difference for the primary outcome: the proportion of patients in corticosteroid-free remission [Harvey‑Bradshaw Index score ≤4] at 12 months at the practice level. The early combined immunosuppression approach was, however, associated with a lower 24-month patient-level composite rate of major adverse outcomes [occurrence of surgery, hospital admission, or serious disease-related complications] versus conventional management [27.7% vs 35.1%, respectively; HR, 0.73; 95% CI, 0.62–0.86; *p* = 0.0003]. As a symptom-based outcome was used in this trial, the effect on outcomes may have been attributable to improved control of inflammation rather than an impact on the disease course. The ongoing REACT2 trial [ClinicalTrials.gov, NCT01698307] may provide some clarification. In this case, the target is mucosal healing rather than symptom control; this trial should offer valuable insight into a T2T approach.

Our literature review identified data describing the relationship between endoscopic and clinical/PRO-based outcomes. In a chart review of 115 children with new-onset UC, completeness of early response was a better predictor of outcomes than baseline disease severity, leading to a proposal of Paediatric Ulcerative Colitis Activity Index [PUCAI] <10 as a feasible treatment goal.^31^ An analysis of data from the EXTend the Safety and Efficacy of Adalimumab Through ENDoscopic Healing [EXTEND] study showed significant predictive effects of endoscopic assessment scores at Week 12 for quality of life outcomes at Week 52.^35^ In an analysis of 3 years of data from the ‘Adalimumab for the Induction and Maintenance of Clinical Remission in Subjects With Crohn’s Disease’ [CHARM] study and its extension [ADHERE], patients who achieved both clinical response and absence of mucosal ulceration maintained better physical and disease-specific quality of life outcomes compared with patients who achieved only one of these outcomes.^36^

### 3.2. The impact of treating to target on economic outcomes

A number of studies have explored the economic impact of different targeted treatment approaches for IBD. One analysis used a decision analytical model to compare the cost effectiveness of two treatment strategies for patients with moderate-to-severe CD initiating treatment with infliximab.^24,25^ In the clinical response arm, dose was escalated in patients who did not achieve clinical response or remission at Year 1, and in the mucosal healing arm, patients with persistence of mucosal ulceration at Year 1 underwent treatment escalation irrespective of clinical symptoms. Cost-effectiveness data were sensitive to the efficacy of therapy; the mucosal healing strategy was both more effective and less costly than the clinical response approach when mucosal healing and clinical response rates reached 45% and 54%, respectively.^25^

The economic implications of different treatment strategies have also been investigated in the UC setting. Saini and colleagues employed a Markov cohort model to simulate three different treatment strategies in patients with newly diagnosed mild to moderate UC [quiescent disease, after induction of remission with 5-ASA agents].^23^ Costs were derived from sources dating from 2006 to 2009. This study suggested that inflammation-targeted treatment [5-ASA therapy only for patients with a positive stool inflammatory marker] was less costly than symptom-targeted treatment [treatment for symptomatic disease flares only] or continuous maintenance treatment for all patients [cumulative per-patient costs of $22,798, $24,378, and $25,621, respectively]. Given that effectiveness [in terms of quality-adjusted life-years] was similar across the three groups, inflammation-targeted treatment was proposed to be the most cost-effective strategy.

Finally, an economic model has been used to evaluate the cost-effectiveness of a tight control strategy versus conventional clinical management, using data from the CALM trial.^79^ This analysis indicated that cost benefits associated with increased remission rates, reduced hospitalisations, and improved quality of life outweighed the increased drug costs, and the tight control approach was deemed the more cost-effective strategy.^79^

## 4. Strategies Supporting a T2T Approach

### 4.1. Tight control

The STRIDE group did not recommend the use of biomarkers as a target, suggesting that these facilitate the monitoring of a patient rather than being a target for treatment *per se*.^13^ However, repeated endoscopic evaluation may not be practical nor acceptable to patients, given their invasive nature and high cost. The use of biomarkers, serving as a surrogate for certain targets, may have clinical value. ‘Tight control’ describes a management approach in which treatment decisions are based on close monitoring of outcome measures such as biomarkers, with the objective of improvement in patient outcomes as a result.

A prospective randomised trial [CALM] assessed the benefits of a tight control strategy as compared with standard clinical management [symptom-driven care] in CD [preliminary data identified by our literature search; since published in full].^16,17,75^ The study included 244 patients with moderately to severely active CD, with most patients being enrolled relatively early in the disease course [median disease duration 1.0 and 0.9 years in the tight control and clinical management groups, respectively]. Treatment was offered in four escalating steps: in the tight control group, escalation was triggered by failure to fulfil success criteria (CD Activity Index [CDAI] <150, CRP <5 mg/L, faecal calprotectin [FC] <250 μg/g, and no prednisone), whereas in the standard care group, the criteria were simply CDAI decrease ≥70 or 100 [at randomisation or post‐randomisation, respectively] or CDAI <200 and no prednisone, both reflecting routine clinical practice.^16,17^ The primary endpoint was CD endoscopic index of severity [CDEIS] <4 and absence of deep ulcers, which was achieved by 45.9% and 30.3% of the tight control and standard clinical management groups, respectively [adjusted risk difference 16.1 [95% CI, 3.9–28.3; *p* = 0.01].^16^ As well as being associated with superior endoscopic and deep remission, the tight control approach was also associated with a lower rate of CD-related hospitalisations compared with standard clinical management [13.2 versus 28.0 events/100 patient-years; *p* = 0.021].^17^ These data suggest that biomarker levels may be used to guide treatment adjustments in order to achieve superior endoscopic and clinical outcomes.

Our literature search identified two other studies in which treatment for IBD was guided by biomarkers. In a study of 91 adults with UC in remission, treatment was guided by the biomarker FC alone.^34^ Patients were randomised to an intervention group, in which 5-ASA dose escalation was triggered by an FC value >300 mg/g, or a control group with no intervention. No significant difference was reported between these two groups in the relapse rate to Month 18. However, the relapse rate was significantly lower in the active intervention than the control group in the subset of patients with FC >300 mg/g [28.6% vs 57.1%; *p* < 0.05]. In a retrospective observational study of 52 patients with IBD with secondary loss of response to infliximab or adalimumab, patients were assessed for treatment response every 6 months in a virtual clinic, and treatment was adjusted according to biomarkers [CRP and FC] and global physician assessment.^18^ This approach allowed intensified anti-TNF therapy to be offered according to patient need, and was associated with recapture of clinical response in 64% of patients enrolled for ≥12 months; 32% were successfully de-escalated back to standard dosing.

Thus, although biomarkers are not currently recognised as targets in the STRIDE recommendations, a tight-control approach based on biomarkers may be an effective strategy for achieving the target, as demonstrated in CALM.^16,17,75^

### 4.2. Coordinated care by a multidisciplinary team

A T2T management strategy requires a holistic approach with all involved parties working towards common treatment targets, which may involve a multidisciplinary team. Although not reflecting direct assessments of a T2T management approach, some studies identified by this review considered the association of multidisciplinary care with outcomes in IBD. For example, Van Deen and colleagues developed an evidence-based multidisciplinary care pathway which was associated with a positive impact on clinical disease activity indices and quality of life scores, allowed individual flexibility, and harmonised care across providers.^19,74^ Similarly, a ‘patient-centred medical home’ providing total care for patients with IBD, incorporating remote monitoring and telemedicine, has been shown to promote improved quality of life and to reduce the frequency of emergency room visits and hospitalisations.^37,38^ A multicentre study in California demonstrated positive effects of coordinated IBD care and remote patient monitoring on IBD-specific outcomes, including less steroid use, fewer emergency visits, and fewer hospitalisations, compared with standard care.^39,40^ The inclusion of other modalities into the care team may help to optimise the T2T approach; for example, intestinal ultrasound may offer a cost effective alternative to colonoscopy for monitoring disease activity, although this has not been demonstrated in a prospective study.^44^

The patient is, of course, a critical member of the multidisciplinary team and their active commitment to their own disease management is particularly important in a T2T setting. The extent to which patients’ engagement in their care impacts on outcomes was reported by a study of 333 patients with mild/moderate UC and 5-ASA treatment.^42^ A ‘web-group’ of patients receiving disease-specific information and coordinating self-care via a website demonstrated improved adherence to acute treatment, shorter relapse duration, and fewer outpatient visits than a standard care group, though frequencies of relapse, hospitalisation, surgery, and adverse events were unaffected. A German study assessing the impact of involving patients in proactively assessing their health and planning their care reported significantly fewer outpatient visits, fewer disease-related problems, and improved self-management skills [health education impact questionnaire scores] compared with standard care.^41^ A Dutch study [published subsequent to our literature search] evaluated a self-management strategy for patients with IBD supported by a telemedicine system [myIBDcoach] that monitors and registers disease activity.^76^ Over 12 months, the study met one of its primary outcomes: patients randomised to this system had significantly fewer outpatient visits to the gastroenterologist or nurse. The telemedicine system also conferred a significant advantage in terms of hospital admissions compared with standard care. There was, however, no significant difference between groups in mean numbers of flares, corticosteroid courses, emergency visits, and surgeries, nor in patient-reported quality of care scores [the other co-primary outcome].^76^

### 4.3. Adherence to management regimens

As indicated above, patient ‘buy-in’ to understand their treatment target[s] is a critical element of a successful T2T approach. Adherence of patients to their prescribed treatment and monitoring regimens is necessary for prescribed treatment adjustments to be effective in working towards target outcomes. A considerable number of studies identified by this literature review have explored the association between patients’ adherence to treatment for IBD and outcomes. Numerous real-world studies have shown that adherence to IBD therapy [5-ASA or biologic] is associated with improved disease course, fewer hospitalisations and emergency visits, better quality of life, and reduced health care use and costs.^54,56–64,66–70,72^ A budget impact model indicated that the once-daily dosing, made possible with multimatrix mesalamine, may be associated with better adherence and reduced health care use and costs compared with other 5-ASA formulations.^73^ Adherence may be improved by displaying information in a patient-friendly way, offering telephone nurse counselling, or individualising care based on patient preferences.^55,65,71^

### 4.4. Therapeutic drug monitoring

The adjustment of treatment to achieve target serum levels of thiopurines and/or biologics is a form of T2T strategy that can help to support the achievement of the ultimate objective, which is to optimise clinical remission and mucosal healing. There has been much discussion of the merit of using therapeutic drug monitoring in the IBD setting, supported by conflicting evidence on the merits of proactive [routine, at predetermined timepoints] versus reactive [in response to suboptimal disease control] monitoring.^88,89^ The American Gastroenterological Association has specifically considered this issue and does not currently recommend proactive monitoring in IBD,^90^ though there is suggestion that it may yet be adopted as standard of care.^91^ Patients with declining serum drug concentrations, and the appearance of anti-drug antibodies before the development of clinical symptoms, may be the best candidates for treatment adjustment to avoid relapse.

The literature review identified a number of controlled assessments of therapeutic drug monitoring. A prospective, randomised, double-blind trial was conducted in 122 biologic-naïve adult patients with active CD, aiming to establish whether maintenance of serum levels of infliximab above 3 μg/mL through proactive therapeutic drug monitoring produced higher rates of clinical and endoscopic remission than symptom-based dosing adjustments [published subsequent to the literature search].^78^ The primary endpoint of sustained corticosteroid-free clinical remission [CDAI < 150] from Weeks 22–54, with no ulcers at Week 54, was achieved by similar proportions of patients in groups where dosing was adjusted on the basis of clinical symptoms and biomarker analysis and/or serum infliximab concentrations [33% and 27%], as in the control group where adjustments were based on clinical symptoms alone [40%; *p* = 0.50 between groups].^78^ A failure to meet the primary endpoint was also observed in a 1-year randomised controlled trial of 263 patients [178 with CD and 85 with UC] with stable responses to maintenance infliximab therapy, in which dose optimisation was based on a target trough concentration of 3–7 μg/mL.^50^ There was no significant difference in the proportion of patients who achieved remission when dosing was based on clinical features or the target trough concentration of infliximab. The rate of relapse was, however, significantly reduced in the concentration-based dosing group [17% vs 7%, respectively; *p* = 0.018].^50^ A follow-up analysis of this trial [published subsequent to the literature search] revealed that rates of hospitalisation, surgery, and steroid use were below 15% in both groups.^77^ The rate of infliximab discontinuation was, however, significantly lower in the first year after the trial ended in the concentration-based dosing group compared with the clinic-based dosing group (2/21 [10%] versus 10/27 [37%], respectively; *p* = 0.04). The difference between these two groups in rate of infliximab discontinuation in the first year, due to loss of response or immunogenicity, was not significant (1/10 [10%] versus 5/11 [45%], respectively; *p* = 0.15).^77^ In a single-blind trial of patients with CD, patients failing infliximab therapy were randomised to receive either infliximab at an increased dosing frequency of 5 mg/kg every 4 weeks, or treatment based on serum concentrations of infliximab and infliximab antibodies according to a predefined algorithm.^51,52^ The study found that individualised treatment according to the algorithm was more cost-effective than standard dose escalation, although the response rates were not significantly different between the groups.

The literature review also identified a number of uncontrolled, ‘real-world’ studies that explored the success of therapeutic drug monitoring strategies. Retrospective studies have demonstrated, unsurprisingly, that rates of mucosal healing/clinical remission in CD and UC are improved by higher serum drug concentrations.^27,28,48^ In one study—a single-centre retrospective cohort study of children aged 5–18 years with IBD—serum infliximab levels during maintenance treatment did not show significant correlation with patient-reported clinical status.^46^ In a prospective observational study of 52 patients [34 CD, 18 UC], who were developing secondary failure to infliximab, reactive therapeutic drug monitoring, strongly predicted the likelihood of achieving mucosal healing following infliximab dose intensification.^49^ Finally, a retrospective cohort analysis of dose-optimisation events based on therapeutic dose monitoring [*n* = 149] or empirical decisions [*n* = 163] demonstrated that the former approach was associated with higher endoscopic remission rates and fewer relapses.^47^

Velayos and colleagues used a decision analytical model to simulate the responses of patients with CD who had become unresponsive to anti-TNF therapy.^53^ The study explored an aspect of therapeutic drug monitoring that involved *a priori* diagnostic testing to target treatment based on the most likely mechanistic cause for loss of response. The study demonstrated that a testing-based strategy yielded rates of remission and response similar to an empirical dose escalation strategy, but was less expensive [$31,870 vs $37,266, respectively]. The testing-based strategy resulted in a higher percentage of surgeries [48% vs 34%] and lower percentage use of high-dose biological therapy [41% vs 54%] than the empirical approach. Support for the cost-effectiveness of treating to an algorithm based on infliximab trough levels and anti-infliximab antibody formation was generated by a small single-centre evaluation of 33 patients with IBD.^43^ This management approach was associated with a 7.4% annual cost reduction compared with baseline.

## 5. Discussion

A T2T approach has been recommended for quite some years for chronic medical conditions such as rheumatoid arthritis, hypertension, and type 2 diabetes mellitus, and is increasingly being explored as a management strategy for other diseases.^92,93^ For example, treating to a target of clinical remission or low disease activity has been extremely successful in reducing joint damage in patients with rheumatoid arthritis.^94^ Treatment targets are typically specific quantitative measures, based on comprehensive, evidence-based, generally accepted target values.^14^

Publication of the STRIDE recommendations for selecting treatment targets in CD and UC represents a first step towards a T2T approach in IBD management. As stated by the STRIDE authors themselves, more data are needed to determine how treating to these targets might alter the course of IBD and impact on patients’ quality of life.^13^ The STRIDE recommendations highlight the importance of both clinical- and PRO-based remission as a treatment target. This patient-centred approach is forward-thinking and may help to avoid the scenario that has been noted in the rheumatoid arthritis setting, where successful achievement of clinical targets has not necessarily been accompanied by similar trends in patient experience.^94^ Although the literature review did not yield prospective data showing a benefit of targeting mucosal or histological healing instead of symptoms in CD or UC, results of a recent long-term extension of the CALM study suggest that mucosal healing is a valid target: early treatment of patients with CD who achieved the target of endoscopic or deep remission after 1 year of intensive treatment were less likely [versus those who did not achieve either definition of remission] to experience disease progression over a median of 3 years.^95^

Nonetheless, there is potential for targets in IBD to develop further with improved understanding. Clarity may be sought, for example, on the extent of endoscopic healing that should be used to define the target. Indeed, the recommendation for ‘complete ulcer disappearance’ in CD may need to be reconsidered. Remission may be an unrealistic target, and is not clearly defined.^96^ Despite not yet being recommended as a target by STRIDE, histological healing was included as a target in the UC study by Bougen and colleagues.^15^ There is a strong push to target histological healing in UC, despite the lack of prospective data to support this strategy. It might be speculated that cross-sectional imaging and/or histological remission could also become a potential future target for CD management.

**Figure 2.**
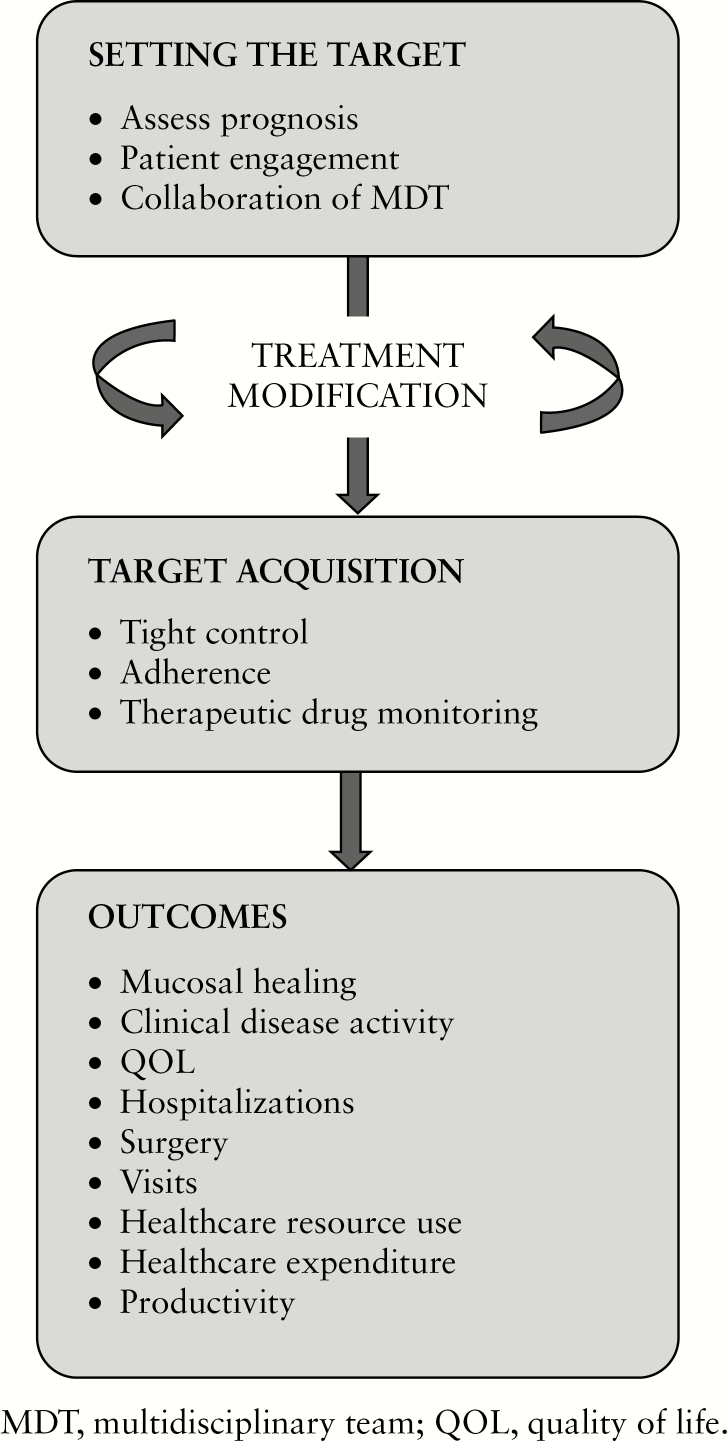
Schematic illustration of factors that may play a role in a treat-to-target strategy in inflammatory bowel disease.

There is a lack of long-term data showing that treating to target in CD or UC is able to block disease progression. Nonetheless, the studies considered in this review indicate that a T2T approach could have a positive impact on clinical and economic outcomes in IBD, in both CD and UC. An overview of factors that may play a role in the T2T strategy is shown schematically in Figure 2. A key strategy supporting the implementation of a T2T approach is that of ‘tight control’. The CALM trial [which has been published in full since the literature review was conducted]^75^ was a T2T study in which the normalisation of biomarkers was part of a tight control strategy. CALM demonstrated a clear benefit, in terms of clinical and endoscopic outcomes, of escalating anti-TNFα and thiopurine therapy at early stages of CD on the basis of measures of disease activity defined by clinical symptoms and biomarkers. It will be useful to evaluate whether there is a ‘window of opportunity’ for therapeutic intervention that is required in order to modify the course of IBD. Delays in initiating disease-modifying antirheumatic drugs [DMARD biologics] are a recognised challenge in efforts to prevent permanent damage from rheumatoid arthritis, for example.^97^

As far as strategies are concerned, the only strong evidence for biomarker normalisation as a part of a tight control approach to achieve the ‘target’ is derived from the CALM study. In real life, the effective use of biomarkers in a T2T approach may be confounded by variation between individual patients in the most appropriate target levels. If the target is mucosal healing, then it is important that the surrogate reflects mucosal inflammation in the individual patient. No recommendation can be made so far to incorporate therapeutic drug monitoring as part of T2T strategies, since the primary endpoints of three prospective studies were not met.^50–52,78^ However, a recent study suggests that proactive [versus reactive] therapeutic drug monitoring of adalimumab in biologic-naïve children with Crohn’s disease was more likely to lead to sustained corticosteroid-free clinical remission [from Week 8 to Week 72],^98^ and there is growing evidence that proactive therapeutic drug monitoring may be a cost-effective strategy.^99^ Nevertheless, the issue is complex: it is quite feasible that optimal serum drug concentrations differ by the target that needs to be reached [such as symptom control, mucosal healing, or fistula repair] and by individual patient factors. Moving forward, it may be increasingly relevant to focus on induction therapy, where factors affecting biologic clearance—such as circulating and tissue TNF levels, low albumin levels, and protein loss—can lead to lower drug concentrations; anti-drug antibodies may also be associated with loss of response. We are getting closer to the point where, using dashboard systems, the pharmacokinetics and early drug clearance can be predicted and could be integrated in the T2T approach.^100^

A strength of the evidence presented in this review is the availability—though very limited—of real-world data. It will be critical that T2T strategies are adequately tested in pragmatic studies, as adopting treatment algorithms in practice can be challenging. A registry study of patients with rheumatoid arthritis demonstrated that although a strategy of treating to a target of clinical remission was considered to be the most effective, direct costs to the health care service were rather high.^101^ It is noteworthy that the study population had established disease and costs were accrued for up to 2 years; as discussed above, this may suggest that the window of opportunity for disease modification had been missed in these patients, and does not negate the possibility that a treat-to-target approach may be more effective over a longer period of time and when considering cost-effectiveness from a broader perspective.

It is relevant to consider the timing of surgery in the context of treating to target in IBD. Traditionally, surgical resection has been reserved for patients with complications and/or refractory disease. This may lead to unnecessary delay in restoration of a better quality of life in cases where response to medical treatment is unlikely. In CD, surgical resection induces ‘mucosal healing’ although intestinal tissue is lost and can lead to unpleasant physiological consequences such as bile acid diarrhoea and vitamin B12 deficiency. In a recent Dutch trial, patients with limited ileal CD refractory to thiopurine treatment were randomised to laparoscopic ileocaecal resection or biologic treatment with infliximab.^102^ One year later, quality of life was similar among both groups, but the surgery approach was economically more beneficial. Hence, it appears that a limited ileocaecal resection, followed by close monitoring of recurrence and treatment intensification accordingly, is a strategy that needs to be considered in suitable patients. Likewise, patients with severe UC who are unlikely to respond to medical treatment could benefit from earlier colectomy, although ideally, predictive markers of [non-]response would be needed to make reliable predictions.

As for any systematic literature review, the search criteria were intended to be inclusive, but may not have considered all relevant publications. For example, the review did not identify the randomised, postoperative Crohn’s endoscopic recurrence [POCER] trial, conducted in a setting which is of considerable interest for the T2T approach. In this trial, patients undergoing intestinal resection of all macroscopic CD lesions were randomised to an active postoperative management strategy which involved stepping up treatment in response to endoscopic recurrence.^103^ The study suggested that selective immune suppression adjusted for early recurrence, rather than routine use, was an effective strategy to prevent postoperative disease recurrence.

The conclusions that may be drawn from this literature review are subject to several limitations. There is a lack of data relating to the potential to slow down disease progression in CD in order to avoid bowel damage and disability; this may be the most relevant long-term outcome. Owing to the paucity of data specifically evaluating T2T approaches in IBD, we considered other aspects of individualised treatment and their links with outcomes. The relevance of these factors to T2T management was variable and not easy to quantify. There is a clear need for longer-term data in order to better evaluate the impact of management strategies on relevant outcomes, particularly in the setting of chronic diseases such as IBD. It should also be considered that T2T is not the only individualised management approach. A modelling exercise suggested, for example, that a benefit-based tailored treatment approach that aimed to reduce the estimated risk of complications based on age, sex, and biomarker values, could prove more effective and cost-efficient than a T2T approach with the aim of achieving target levels of biomarkers in patients with type 2 diabetes.^104^

## 6. Conclusions

As the movement towards treating to target in IBD gains momentum, it is timely to consider the available evidence supporting its implementation in practice and to initiate a number of research initiatives that will answer important remaining questions. Studies have indicated that a T2T approach can positively impact on clinical, economic, and patient-centred outcomes in CD and UC. Longer-term data are, however, currently lacking; the extent to which the potential benefits are restricted to early stages of disease needs to be further defined. In support of these findings, aspects of management such as multidisciplinary care and patient engagement and adherence have been seen to contribute positively to outcomes in IBD, further emphasising the emerging role of individualised care. Stronger evidence of long-term cost-effective benefits is needed in order to implement T2T strategies in routine practice and to shift current practices.

## Funding

This work [systematic literature review and medical writing support] was funded by AbbVie Inc.

## Conflict of Interest

JFC reports grants and personal fees from Abbvie, personal fees from Amgen, Boehringer-Ingelheim, Cellgene Corporation, Celltrion, Enterome, Ferring, Genetech, Medimmune Merck & Co., Pfizer, Protagonist, Second Genome, Seres, Shire, Theradiag, Intestinal Biotech Development, and Genefit; grants and personal fees from Janssen and Janssen, Takeda, outside the submitted work. GRDH has served as adviser for Abbvie, Ablynx, Allergan, Amakem, Amgen, AM Pharma, Arena Pharmaceuticals, AstraZeneca, Avaxia, Biogen, Bristol-Myers Squibb, Boehringer Ingelheim, Celgene/Receptos, Celltrion, Cosmo, Covidien/Medtronics, Ferring, DrFALK Pharma, Eli Lilly, Engene, Galapagos, Genentech/Roche, Gilead, GlaxoSmithKline, Hospira/Pfizer, Immunic, Johnson and Johnson, Lycera, Medimetrics, Millenium/Takeda, Mitsubishi Pharma, Merck Sharp & Dohme, Mundipharma, Nextbiotics, Novonordisk, Otsuka, Pfizer/Hospira, Photopill, Prometheus Laboratories/Nestle, Progenity, Protagonist, Robarts Clinical Trials, Salix, Samsung Bioepis, Sandoz, Seres/Nestle, Setpoint, Shire, Teva, Tigenix, Tillotts, Topivert, Versant and Vifor; received speaker fees from Abbvie, Biogen, Ferring, Johnson and Johnson, Merck Sharp & Dohme, Mundipharma, Norgine, Pfizer, Samsung Bioepis, Shire, Millenium/Takeda, Tillotts and Vifor, outside the submitted work. RP reports grants and personal fees from AbbVie, outside the submitted work. W-JL and JP are AbbVie employees and may own AbbVie stock and/or options.
